# Supplementary material for: Complexome profiling of the Chlamydomonas psb28 mutant reveals TEF5 as an early PSII assembly factor
Source: Plant Cell. 2025 Mar 17;37(6):koaf055. doi: 10.1093/plcell/koaf055 (PMC12164590; doi:10.1093/plcell/koaf055)
Supplement: koaf055_Supplementary_Data [file koaf055_supplementary_data.zip › psb28_interactive.zip!/plots/intensity/qualityPlots/peptides/PRPS17_Cre02.g118950.t1.2.html]

### Cre02.g118950.t1.2

Trivial name: PRPS17  
SeqCoverage [median]: 35.20  
Mass: 11.74 kDa  
  
 

| Sequence | MissedCleavage | Mass [Da] | wtIntensity | muIntensity | Score |
| --- | --- | --- | --- | --- | --- |
| FSVTEVLR | 0 | 949.52 | 0.00 | 1381.11 | 74.13 |
| LATDVTYQK | 0 | 1037.54 | 0.00 | 697.43 | 60.44 |
| LATDVTYQKR | 1 | 1193.64 | 0.00 | 308.27 | 41.40 |
| TVVVEAER | 0 | 901.49 | 0.00 | 425.93 | 65.25 |
| YMAHDESGALR | 0 | 1248.56 | 0.00 | 1134.80 | 85.96 |
|  |  |  | 0.0 | 3947.5 |  |
